# Recent advances in management of acalculous cholecystitis

**DOI:** 10.12688/f1000research.14886.1

**Published:** 2018-10-18

**Authors:** Bryan Balmadrid

**Affiliations:** 1 Department of Gastroenterology, University of Washington Harborview Medical Center Campus, Seattle, USA

**Keywords:** acalculous cholecystitis, EUS, cholecystostomy

## Abstract

Acalculous cholecystitis is a life-threatening gallbladder infection that typically affects the critically ill. A late diagnosis can have devastating outcomes because of the high risk of gallbladder perforation if untreated. The diagnosis is not straightforward as Murphy’s sign is difficult to illicit in the critically ill and many imaging findings are either insensitive or non-specific. This article reviews the current imaging literature to improve the interpretation of findings. Management involves a percutaneous cholecystostomy, surgical cholecystectomy, or more recently an endoscopically placed metal stent through the gastrointestinal tract into the gallbladder. This article reviews the current literature assessing the outcomes of each treatment option and suggests a protocol in determining the modality of choice on the basis of patient population. Specifically, endoscopic ultrasound-guided gallbladder drainage is a novel drainage approach for patients who are poor candidates for surgery and obviates the need for a percutaneous drain and all its complications. It has promising results but has caveats in its uses.

## Introduction

Acalculous cholecystitis is an uncommon but potentially devastating infection of the gallbladder. The diagnosis can be difficult to make, but early recognition is important. The established treatment options have shown success, and a new treatment modality has shown promise. However, the exact order and preference of the treatment modalities remain unclear. This article will summarize the recent literature and discuss factors that determine the appropriate treatment modality.

The gallbladder acts a reservoir for 30 to 50 mL of highly concentrated bile acids, which are necessary in dietary lipid absorption. Gallbladder contractions occur during meals, releasing more bile acids into the duodenum to help absorb the food ^1^. Gallbladder dysmotility or stasis combined with high cholesterol states can cause gallstone formation, potentially leading to blockage of the cystic duct and causing acute cholecystitis. Gallstones cause the majority of acute cholecystitis. Non-gallstone or acalculous cholecystitis occurs less frequently and is often overlooked, leading to a delayed diagnosis.

Acalculous cholecystitis is a gallbladder infection not related to gallstones, leading to serious consequences. It accounts for only 10% of acute cholecystitis but has higher morbidity and mortality than calculous cholecystitis ^2^ since these patients are typically sicker at baseline.

As with many infections, the mainstay of treatment is antibiotics and source control, and the latter typically involves a cholecystectomy or a percutaneous drain into the gallbladder. With critically ill patients, the order or type of treatment is less clear. It is common to place a percutaneous cholecystostomy (PC) for unstable patients, but the question arises whether this can be a destination therapy or whether a cholecystectomy should be performed when more stable. A new modality of internal drainage within the gastrointestinal (GI) tract has arisen from the literature but its role is still being debated. We will discuss new studies that address these issues in detail.

## Acalculous cholecystitis

Acalculous cholecystitis is caused by gallbladder stasis from hypomotility that leads to increased intraluminal pressures in the gallbladder wall, resulting in ischemia, inflammation, and potential necrosis. As with any stasis, it can lead to bacterial colonization and progress to gallbladder infection. Continued ischemia, inflammation, or infection (or a combination of these) can result in perforation, which occurs in about 10% ^2^. These people are often critically ill prior to developing acalculous cholecystitis and thus these complications can be devastating. Significant illness and stressors more commonly found in patients in the intensive care unit can cause gallbladder dysmotility. Stroke, heart attack, severe burns, trauma, major surgeries, and prolonged total parenteral nutrition use have all been associated with acalculous cholecystitis. Table 1 lists more disease associations. It is not surprising then to see historically high mortality rates of 30% ^2^. Note that this diagnosis is not limited to sick patients in a hospital setting. In one smaller retrospective study of 47 patients, the majority (72%) of patients developed symptoms of acalculous cholecystitis in the outpatient setting ^3^.

**Table 1.** Diseases associated with acalculous cholecystitis.

Stress	Comorbidities	Infection-related	Miscellaneous
Trauma	Diabetes mellitus	Salmonella	Systemic lupus
Burns	End-stage renal disease	*Staphylococcus aureus*	Vasculitides
Cardiopulmonary resuscitation	Congestive heart failure/ coronary artery disease	Cytomegalovirus	
Sepsis	Peripheral vascular disease		
Total parenteral nutrition		Immunosuppression	
Mechanical ventilation		AIDS	
Bone marrow/ stem cell transplant		Microsporidia/ cryptosporidium	
Major surgeries			

## Presentation and diagnosis

In the ambulatory patients, the presentation is similar to that of calculous cholecystitis, in which there is right upper quadrant pain, fever, and a positive Murphy’s sign. However, the diagnosis can be more difficult in the critically ill as it may present with non-specific but serious symptoms of sepsis, change in mental status, and overall worsening of the clinical course ^4^. The patient may not be able to verbalize abdominal discomfort. Acalculous cholecystitis typically affects males of older age compared with its gallstone counterpart, in which there is a female predominance for calculous cholecystitis ^5^. There are typically mild elevations in the liver function tests. However, acalculous cholecystitis does not directly cause jaundice, at least not in the early stages of the disease. Sepsis-related cholestasis or, more rarely, an anatomical compression of the common bile duct from a dilated gallbladder (Mirizzi syndrome) can eventually lead to jaundice.

A combination of ultrasound imaging and cholecintigraphy with cholecystokinin (HIDA-CCK) can confirm the diagnosis. Many times, an ultrasound showing a distended gallbladder with a thickened wall and inflammation without stones can be diagnostic. A highly thickened wall or the development of pericholecystic fluid increases the specificity. It is important to note that although an ultrasound alone is sensitive for this diagnosis ^6^, critically ill patients often have abnormal ultrasound findings in the gallbladder without having acalculous cholecystitis ^7^, decreasing the specificity for ultrasounds.

Computed tomography (CT) imaging has high sensitivity similar to that of an ultrasound but lacks specificity. Critically ill patients have a higher frequency of gallbladder abnormalities on CT compared with ultrasound. In a large, case-controlled, retrospective study (n = 127 cases) specifically studying acalculous cholecystitis in critical care units, 96% of critically ill patients had abnormal gallbladder findings on their CT images. These findings include increased thickness and lack of enhancement of the gallbladder wall, subserosal edema, increased bile density, large perpendicular diameters of the gallbladder, gas within the gallbladder, ascites, peritoneal fat edema, and diffuse tissue edema. (See Table 2 for further CT findings with the sensitivity and specificity if available.) The most specific finding for acalculous cholecystitis was gas in the gallbladder with specificity of 99.2% but a very low sensitivity of 11.1%. Alternatively, lack of any gallbladder findings has a very good negative predictive value, effectively ruling out acalculous cholecystitis ^8^. Only nine of the 43 cases with presumed acalculous cholecystitis had a necrotic gallbladder on post-cholecystectomy pathology. This fact limits the accuracy of their proposed sensitivities and specificities.

**Table 2.** Computed tomography findings associated with acalculous cholecystitis.

Findings	Specificity for necrotic GB, percentage	Sensitivity for necrotic GB, percentage
Gas within the GB	99.2	11.1
Lack of GB wall enhancement	94.9	37.5
Subserosal edema	92.4	22.2
Thickness and enhancement of the GB wall	NA	25
High-density bile	NA	13
Increased perpendicular diameters of the GB	NA	78
Peritoneal fat edema	NA	89
Diffuse tissue edema	NA	89
Ascites	NA	100

GB, gallbladder; NA, not applicable.

In the HIDA-CCK, the CCK causes the gallbladder to contract and then an ejection fraction is measured. As is the nature of nuclear studies, this test can take hours to perform and thus is appropriate in only select patients. An ejection fraction of less than 35% is indicative of gallbladder dysfunction and thus acalculous cholecystitis. The sensitivity and specificity range between 67 and 100% and between 58 and 88%, respectively ^9^.

There are several other studies and meta-analysis assessing the sensitivity and specific of imaging modalities in diagnosing acute cholecystitis. However, most exclude acalculous cholecystitis from the analysis. There are imaging criteria for the diagnosis of acalculous cholecystitis available in the literature, such as a review from Barie and Eachempati ^10^. The important findings to look for in imaging are a distended gallbladder without stones along with a thickened or edematous wall. Further findings serve to improve the specificity. As always, incorporate other clinical data to make the diagnosis.

## Treatment

Administration of intravenous antibiotics plays the first role in treatment for acalculous cholecystitis in the hospital setting. The Surgical Infection Society and the Infectious Diseases Society of America provided guidelines in 2010 that base their antibiotic recommendations on the whether this is community- or hospital-acquired, but we will focus on the hospital-acquired regimens. For monotherapy, the carbapenems and piperacillin/tazobactam are sound options. For other regimens, including ones that take into account extended-spectrum beta-lactamase (ESBL)-producing organisms, see Table 3 ^11^. The duration of antibiotics depends on source control and can be stopped four to five days after this is achieved ^11, 12^. In the difficult situation in which source control cannot be achieved, the antibiotic regimen should be based on decreasing inflammatory markers, resolution of fevers, and improvement in clinical condition. In this situation, studies and official recommendations are lacking and thus clinicians should consider the duration of antibiotics on a case-by-case basis.

**Table 3.** Antibiotic agents for initial empiric treatment of acalculous cholecystitis.

Situation	Regimen
Mild to moderate infection	Cefazolin, cefuroxime, and ceftriaxone
Severe infection or high-risk factors such as advanced age, immunocompromise, and end-organ disease	Imipenem-cilastatin, meropenem, doripenem, piperacillin-tazobactam, ciprofloxacin, levofloxacin, or cefepime, each in combination with metronidazole
Extended-spectrum beta-lactamase (ESBL)-producing organisms	Imipenem-cilastatin, meropenem, doripenem, and piperacillin-tazobactam, each in combination with metronidazole
Health care–associated infection of any severity	Add vancomycin to appropriate regimen above.

Adapted from the Surgical Infection Society and the Infectious Diseases Society of America guidelines of 2010 ^11^.

The traditional treatment for source control has been a PC or surgery. Transpapillary drainage through an endoscopic retrograde cholangiopancreatography (ERCP) has been used with variable success and suffers from high recurrence rates ^13^. We will not be discussing this option in further detail. The treatment of choice would be a surgical cholecystectomy. However, many of these patients are in critical condition and are poor surgical candidates. This is when a PC tube placed by interventional radiology to secure gallbladder drainage is used. This can act as both a temporizing measure (bridge to surgery) and a treatment option. One very large retrospective study of 1,725 cases suggests that, in extremely ill patients, a PC has lower morbidity, fewer intensive care unit admissions, decreased length of stay, and lower costs compared with open cholecystectomy ^14^. Acute complications for PC tube remain low with an overall rate of around 2% ^15, 16^. Keep in mind that patients who get percutaneous drainage are typically sicker with higher mortality rates overall and mortality may not be directly related to the intervention itself. Thus, the common practice involves an initial PC in any high-risk surgery patients.

The question arises of who should undergo an attempt of a surgical cholecystectomy after stabilization with a PC. In a recent retrospective review of 271 patients with a PC for acalculous cholecystitis, 46.8% of patients had interval cholecystectomy mostly during the index admission. There was an 8.5% 30-day mortality. The patients who died in the hospital were excluded from the outcomes evaluation, skewing the conclusions. In the remaining 44.6% (121 patients) who were treated with only a PC, the percutaneous drain was removed successfully in 72.7% following a successful trial of catheter clamping whereas the rest had the catheter remain. The recurrence rate after removal of the drain was only 2.3% ^15^. This is one of the largest studies specifically looking at initial PC treatment for acalculous cholecystitis. It reinforces that PC is safe and effective, but it also highlights that this can be the lone treatment with a good rate of eventually removing the tube. In patients who are not good candidates for surgery in whatever stage of the disease, percutaneous drainage may be enough for treatment. Its retrospective nature reduces the strength of this study conclusion.

In terms of the timing of drain removal, there is no consensus. A sensible approach is to first wait for resolution of clinical symptoms such as fever or leukocytosis. Then a week after resolution, a cholecystogram should be performed, and if the cystic duct is patent and contrast empties readily into the duodenum, then these patients are candidates for removal of the PC tube.

**Figure 1.**
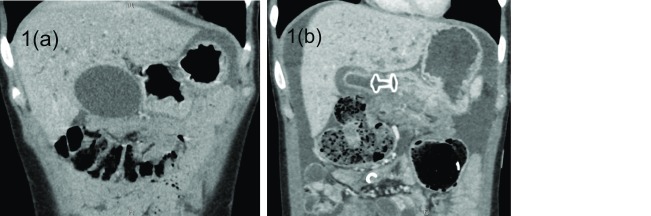
Lumen-apposing metal stent placement in the gallbladder. ( **a**) This computed tomography image shows a distended gallbladder in close proximity to the antrum of the stomach. ( **b**) This fully covered lumen-apposing metal stent is creating communication between the gastric antrum and the now-significantly-decompressed gallbladder.

Endoscopically placing a lumen-apposing fully covered metal stent (LAMS) through the GI tract into the gallbladder has emerged as a new and viable alternative for drainage. This is performed with an ultrasound endoscope. Through ultrasound guidance, the deployment device punctures through the duodenal bulb or gastric antrum to enter the gallbladder. The two anchoring flanges of the stent deploy in the gallbladder and the GI tract to create a secure conduit between the two ( Figure 1). This method of internal drainage obviates the need for a percutaneous drain along with its disadvantages. Drains often cause patient discomfort, have a risk of dislodgement, and require daily drain maintenance. A multicenter retrospective review compared endoscopic ultrasound-guided gallbladder drainage (EUS-GBD) with LAMS to a PC in 90 patients with either calculous cholecystitis (n = 61) or acalculous cholecystitis (n = 29) ^17^. The data showed similar very high technical and clinical success with similar low adverse events between 11 and 14%. This study did not provide the power for the specific complications, but there were two episodes of bleeding in the EUS-GBD group and none in the PC group. Alternatively, there was one bile leak in the EUS-GBD group and three in the PC group. What was significant is the low rate of re-intervention per patient in the EUS-GBD group of 0.2 compared with 2.5 interventions per patient in the PC group. There was no stent migration in the EUS-GBD group ^17^. Based on a recent systematic review of 189 cases, the overall stent migration rate when used in the gallbladder is low (1.1%) ^18^. The EUS-GBD group had lower pain scores, shorter hospital stays, and fewer repeated interventions ^17^, making this intervention an attractive alternative to treatment. The articles had the same limitations of any study evaluating new techniques. This is a retrospective study performed only by experts, and the overall number of cases using the EUS-GBD approach is low (45) and if only acalculous cholecystectomy is taken into account, that number drops to 18. However, this is one of the largest studies available in comparing EUS-GBD with PC. A similar retrospective study performed in one center without a comparison group showed similar results and adverse events. This study had 75 total patients, of whom 18 had acalculous cholecystitis ^19^. Similar to the other study, there is no subset analysis of the acalculous cholecystitis group and thus conclusions are extrapolated to this specific subset, greatly limiting its strength of evidence.

It would be helpful to have a prospective study directly comparing the EUS-GB approach to PC in patients specifically deemed to be poor surgical candidates in the acute cholecystitis setting and specifically plan to perform subset analysis on acalculous cholecystitis. It is important to note that EUS-GBD should be reserved for patients not expected to ever undergo surgery. Approximating the gallbladder to the duodenal bulb increases the difficulty and risk of surgically removing the gallbladder as it can lead to duodenal or gastric perforation since there is a 15-mm luminal defect created by the LAMS. Thus, surgery is avoided in this situation. Although technically removable, these metal stents were meant to be in place permanently in this situation. In comparison, a percutaneous tube can act as a bridge and does not add risk to a surgical cholecystectomy, the most definite treatment.

## Summary

Consider the diagnosis of acalculous cholecystitis in critically ill patients with worsening condition of unclear etiology. A late diagnosis can be devastating as it often leads to perforation and sepsis. Unfortunately, there are no clear diagnostic criteria. In these patients, first rule out common causes of infection. Then look for risk factors in Table 1 in combination with imaging studies and lab values to make the diagnosis. There are several non-specific, yet sensitive signs on CT and ultrasound starting with gallbladder distension and a thickened or edematous gallbladder. Although gas in the gallbladder clinches the diagnosis, one should not wait for this insensitive finding. The sicker the patient, the lower the threshold to make the diagnosis and treat acalculous cholecystitis. In sicker patients not amendable to surgery, a PC is a good first option and may be the single modality of treatment. There is also a high likelihood to remove the percutaneous drain eventually. This is especially helpful for patients as drain discomfort, drain care, and the risk of drain dislodgement can affect their quality of life. In select patients, the percutaneous approach can be avoided completely. Based on a recent study, EUS-GBD with LAMS has shown equivocal success and adverse events compared with a PC while avoiding a percutaneous drain and improving overall patient comfort. However, this should be reserved specifically for patients who are deemed to not ever be a surgical candidate and have shorter life expectancy or poor radiological windows for PC or have ascites. There have been reported issues with long-term stent migration, perforation from the stent, and tissue ingrowth leading to obstruction within the stent, but the overall complication rate is low. An ERCP with transpapillary drainage through the cystic duct should be the last option. Prospective studies with specific protocols addressing EUS-GBD candidacy and LAMS post-procedure management would better highlight its role in acalculous cholecystitis. Following this, results of a randomized control study comparing all the different modalities would allow an updated management protocol for these sick patients.

## Abbreviations

CT, computed tomography; ERCP, endoscopic retrograde cholangiopancreatography; EUS-GBD, endoscopic ultrasound-guided gallbladder drainage; GI, gastrointestinal; HIDA-CCK, cholecintigraphy with cholecystokinin; LAMS, lumen-apposing fully covered metal stent; PC, percutaneous cholecystostomy.
